# Placental oxygen transport estimated by the hyperoxic placental BOLD MRI response

**DOI:** 10.14814/phy2.12582

**Published:** 2015-10-14

**Authors:** Anne Sørensen, Marianne Sinding, David A Peters, Astrid Petersen, Jens B Frøkjær, Ole B Christiansen, Niels Uldbjerg

**Affiliations:** 1 Obstetrics and Gýnecology, Aalborg University HospitalAalborg, Denmark; 2 Clinical Engineering, Aarhus University HospitalAarhus, Denmark; 3 Pathology, Aalborg University HospitalAalborg, Denmark; 4 Radiology, Aalborg University HospitalAalborg, Denmark; 5 Obstetrics and Gynecology, Aarhus University HospitalAarhus, Denmark

**Keywords:** Placental oxygen transport, placental MRI, placental funktion, fetal growth restriction

## Abstract

Estimating placental oxygen transport capacity is highly desirable, as impaired placental function is associated with fetal growth restriction (FGR) and poor neonatal outcome. In clinical obstetrics, a noninvasive method to estimate the placental oxygen transport is not available, and the current methods focus on fetal well-being rather than on direct assessment of placental function. In this article, we aim to estimate the placental oxygen transport using the hyperoxic placental blood oxygen level-dependent (BOLD) magnetic resonance imaging (MRI) response. In 21 normal pregnancies and in four cases of severe early onset FGR, placental BOLD MRI was performed in a 1.5 Tesla MRI system (TR:8000 msec, TE:50 msec, Flip angle:90). Placental histological examination was performed in the FGR cases. In normal pregnancies, the average hyperoxic placental BOLD response was 12.6 ± 5.4% (mean ± SD). In the FGR cases, the hyperoxic BOLD response was abnormal only in cases with histological signs of maternal hypoperfusion of the placenta. The hyperoxic placental BOLD response is mainly derived from an increase in the saturation of maternal venous blood. In the normal placenta, the pO_2_ of the umbilical vein is closely related to the pO_2_ of the uterine vein. Therefore, the hyperoxic placental BOLD response may reflect the placental oxygen supply to the fetus. In early onset FGR, the placental oxygen transport is reduced mainly because of the maternal hypoperfusion, and in these cases the placental BOLD response might be altered. Thus, the placental BOLD MRI might provide direct noninvasive assessment of placental oxygen transport.

## Introduction

Estimating placental oxygen transport capacity is highly desirable, as impaired placental function is associated with fetal growth restriction (FGR) and poor neonatal outcome (Bernstein et al. 2000; Baschat 2011) because of increased risk of fetal hypoxia and acidosis (Nicolaides et al. 1989). Already in 1946, it was stated by Sir Joseph Barcroft in the Researches on Pre-natal Life, that; “oxygen crosses the placental membrane by simple diffusion, and the transport is driven toward the fetus by the partial pressure gradient of oxygen” (Barcroft 1946). Later, it has been demonstrated that the fetal oxygen supply is determined by the oxygen diffusion across the placental membrane and the blood oxygen content and blood flow of the uterine and umbilical vessels (Carter 1999). In early onset FGR (before 34th week of gestation), placental oxygen transport is reduced mainly because of maternal hypoperfusion (Lin et al. 1995) due to abnormal transformation of the spiral arteries into low-resistance vessels (Chaddha et al. 2004).

In clinical obstetrics, a direct noninvasive test of placental oxygen transport is not available and current methods such as ultrasound Doppler measurements of fetal and umbilical blood flow estimate fetal well-being rather than the placental function. A timeline of changes in fetal circulation has been described in relation to the progression of fetal distress from hypoxia into acidosis and still birth (Baschat 2005). In modern obstetrics, these circulatory changes in combination with fetal heart rate monitoring are the main indicators of when to deliver the growth-restricted fetus. Doppler flow measurement of the uterine arteries is the only clinical method to evaluate the placental perfusion in vivo. The pulsatility index (PI) of the uterine arteries reflects the downstream resistance of the maternal side of the placental vascular bed, which is mainly determined by the resistance of the spiral arteries (Figueras and Gardosi 2011). In the first trimester, Doppler flow examination of the uterine arteries is a valuable screening tool for early onset of FGR (Pilalis et al. 2007) and pre-eclampsia (Poon et al. 2009), and in the third trimester, persistence of the increased resistance of the uterine arteries is an indicator of adverse neonatal outcome in the growth-restricted fetus (Ghi et al. 2010). However, uterine Doppler flow does not estimate the placental function directly, nor does the PI correlate with the oxygen transport capacity. Therefore, new methods are highly needed in this field.

Blood oxygen level-dependent (BOLD) magnetic resonance imaging (MRI) provides noninvasive information of changes in tissue oxygenation. The relation between the BOLD signal and tissue oxygenation relies on the paramagnetic properties of deoxyhemoglobin. The presence of deoxyhemoglobin creates magnetic field inhomogeneities, thereby reducing the BOLD MR signal (Logothetis and Pfeuffer 2004). In previous literature, by using invasive measurements of fetal oxygenation in a sheep model, it has been demonstrated that changes in BOLD signal reflects changes in fetal oxygenation (Wedegartner et al. 2006; Sorensen et al. 2009). Furthermore, in human pregnancy, by using the noninvasive BOLD method, it has been demonstrated that hyperoxia increases fetal and placental oxygenation (Sorensen et al. 2013a,b). In the placental, the majority of deoxyhemoglobin is of maternal origin, as the amount of fetal blood in the placenta is relatively small. Furthermore, as maternal arterial blood is already fully saturated at room air, the placental hyperoxic BOLD response is derived predominantly from an increase in the saturation of maternal venous blood. The pO_2_ of the fetal umbilical vein is closely related to the pO_2_ of the uterine vein (Pardi et al. 1992)and therefore the hyperoxic placental BOLD response may reflect the placental oxygen supply for the fetus.

In this article, the hyperoxic placental BOLD response was investigated in 21 normal pregnancies and in four cases of severe FGR. We hypothesize that the hyperoxic placental BOLD response reflects the placental oxygen transport. To the best of our knowledge, this is the first study to investigate the hyperoxic placental BOLD response in human FGR pregnancies.

## Methods

### Ethical approval

Informed oral and written consent was obtained from all participants in accordance with the standard defined in the Declaration of Helsinki. The study was approved by the Regional Committee on Biomedical Research Ethics (Journal number M-20090006 and N-20090052).

### Subjects

In the normal group, we included 21 healthy pregnant women carrying uncomplicated singleton pregnancies in gestational week 24–40. Ultrasound estimated that the fetal weight was normal and Doppler flow measurements of the umbilical artery, the middle cerebral artery, and the maternal uterine arteries were also within the normal range. A subset of the normal group (*n* = 8) was already used in a previous publication by our group (Sorensen et al. 2013a). In the FGR group, we included four women carrying singleton fetuses with estimated fetal weight below the 1st centile. The individual characteristics of each FGR case including ultrasound Doppler flow measurements are presented in Table1. Case 4 had normal Doppler measurements. The remaining three cases demonstrated abnormal Doppler flow in the uterine arteries. Furthermore, the fetal cerebral blood flow was increased as a result of redistribution of fetal blood flow. In Case 1, even the ductus venosus blood flow was abnormal, which is a sign of diastolic cardiac failure.

**Table 1:** FGR case characteristics

FGR	GA at MRI:	EFW:	Doppler findings (*Z*-scores):	ΔBOLD (%)	Outcome:	Birth weigth:	Placental examination:
CASE 1	24 + 5	361 g (−51%)	CPR 0.40 (−4.26)	0.1	Still birth Vaginal delivery	380 g	Low weight 93 g (−59%)
			DV flow PI 1.63 (6.85)		GA = 26 + 0		Normal shape
			UtA flow Mean PI 1.69 (1.89)				Decidual!arteropathy (atherosis)
							Accellerated maturation
							Distal villous hypoplasia!
							Increased intervillous fibrin deposition
							Multiple infarcts (65%)
CASE 2	29 + 0	962 g (−31%)	CPR 0.58 (−4.01)	26.8	Good	974 g	Low weight 185 g (−37%)
			DV flow PI 0.77 (1.36)		Acute CS		Abnormal shape (“pancake”)
			UtA flow Mean PI 1.90 (2.58)		GA = 29 + 2		Accellerated maturation
							Distal villous hypoplasia
							Multiple infarcts (50%)
CASE 3	30 + 0	956 g (−43%)	CPR 0.89 (−3.28)	29.5	Good	1050 g	Low weight 191 g (−44%)
			DV flow PI 0.73 (1.21)		Acute CS		Abnormal shape (“cupcake”)
			UtA flow Mean PI 2.37 (3.43)		GA = 31 + 0		Accellerated maturation
							Distal villous hypoplasia
							Few infarcts (5%)
							Marginal cord insertion
							Fetal thrombotic vasculopathy
CASE 4	31 + 1	1094 g (−39%)	CPR 1.44 (−1.85)	13.4	Good	1515 g	Low weight, 267 g (−35%)
			DV flow PI 0.54 (0.01)		Elective CS		Normal shape
			UtA flow Mean PI 0.77 (−0.54)		GA = 34 + 0		Normal histology

GA, gestational age (week); EFW, estimated fetal weigth; CPR, cerebroplacental ratio; DV, ductus venosus; UtA, uterine artery; PI, pulsatility index; CS, cesarean sectio.

### MRI protocol

The BOLD MRI scan was performed in a GE Discovery MR450 1.5 Tesla MRI System (GE Healthcare, Milwaukee). An eight channel cardiac coil was positioned over the abdomen covering the uterus. Data were acquired with a gradient echo-planar imaging sequence with the following parameters: repetition time = 8000 msec, echo time (effective) = 50 msec, flip angle = 90, and 22 slices of 6 mm with a slice gap of 6 mm. Field-of-view of 36 × 36 cm with a matrix of 128 × 128 resulted in an in-plane spatial resolution of 3.6 × 3.6 mm. A total of 90 dynamics were acquired in each scan.

During the BOLD MRI scan, the maternal oxygen supply was controlled by a non-rebreather facial mask (Hudson Respiratory Care, Durham, NC). Two five-minute episodes of different oxygenation were recorded: the initial normoxic episode followed by the hyperoxic episode (12 L 100% O_2_ per minute). The facial mask was applied without interfering with the BOLD MRI scan while the pregnant woman remained in the bore magnet in the left lateral position.

### MRI analysis

The BOLD MRI DICOM data were processed using an in-house developed program written in MATLAB (The MathWorks Inc, Natick, MA). Three central slices showing cross sections of the placenta with a distance of 30 mm were investigated. Regions of interest (ROIs) were drawn covering the entire placenta, and the location of the ROI was adjusted in each dynamic image, correcting for maternal breathing movements. All ROIs were drawn by a single observer (AS).

### Statistical analysis

The BOLD signal in each placenta was calculated in each dynamic image as an average of the three placenta ROIs (one from each slice). For each placenta, the changes in BOLD signal were estimated as a percentage of the initial three minutes of the normoxic phase. Changes in BOLD signal versus time plots were drawn for each of the FGR cases and for the group of normal controls. Furthermore, the hyperoxic BOLD response (ΔBOLD) was calculated as the mean percentage increase in BOLD signal of the last two minutes of the hyperoxic phase (the steady-state level). In the group of normal controls, the mean increase in BOLD signal was tested using a paired student’s *t*-test and a *P*-value below 0.05 was considered statistically significant. The paired differences in placental BOLD signal were tested and found to be normally distributed.

### Postpartum placental examination

In each FGR case, a placental examination was performed by an experienced perinatal pathologist (AP). All placentas were examined according to a standard protocol which included “trimmed” weight, measurement of area and thickness, and histological examination by light microscopy of standard sections (central placental parenchyma, placental parenchyma at the umbilical cord insertion, umbilical cord, membranes, and sections from macroscopic abnormalities) (Redline et al. 2004). Estimated placental and fetal weights were correlated with normal reference values (Marsal et al. 1996; Pinar et al. 1996). The histological examination included assessment of villous maturation, description of microscopic abnormalities, and description of the fetal and maternal vasculature.

## Results

**Figure 1:**
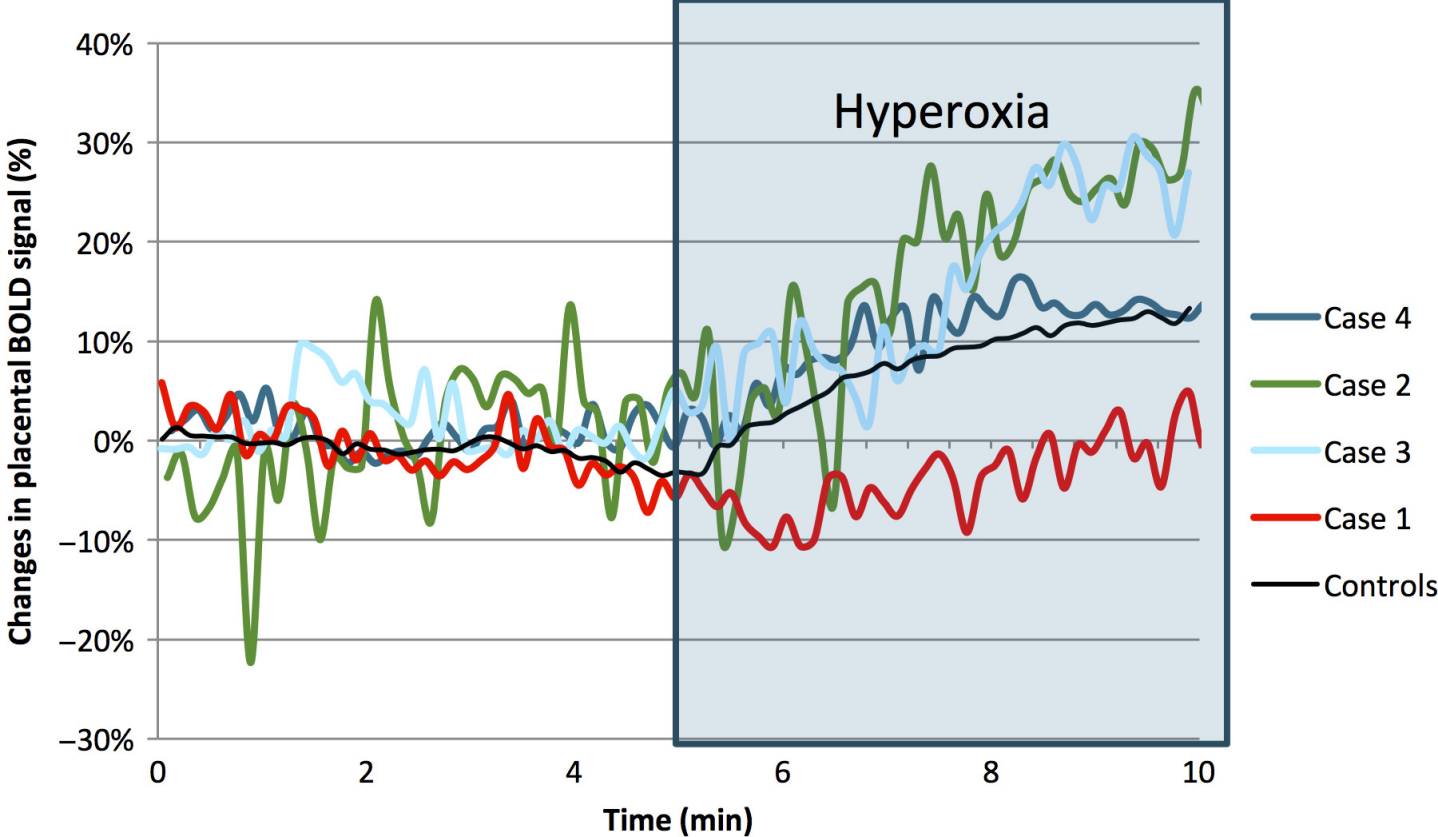
The hyperoxic placental BOLD response (0BOLD) in each of the four FGR cases, and a mean value of normal controls (*n* = 21).

In the normal group, the average hyperoxic placental BOLD response was 12.6 ± 5.4% (mean ± SD), this increase in highly significant (*P* < 0.01). The placental BOLD response was normal in FGR Case 4 (ΔBOLD = 13.4%); however, in the remaining three FGR cases, the BOLD response was abnormal. The BOLD response was above normal in Case 3 (ΔBOLD = 29.5%) and Case 2 (ΔBOLD = 26.8%). In Case 1, there was no increase in placenta BOLD signal during maternal hyperoxia (ΔBOLD = 0.1%). (Fig.1).

**Figure 2:**
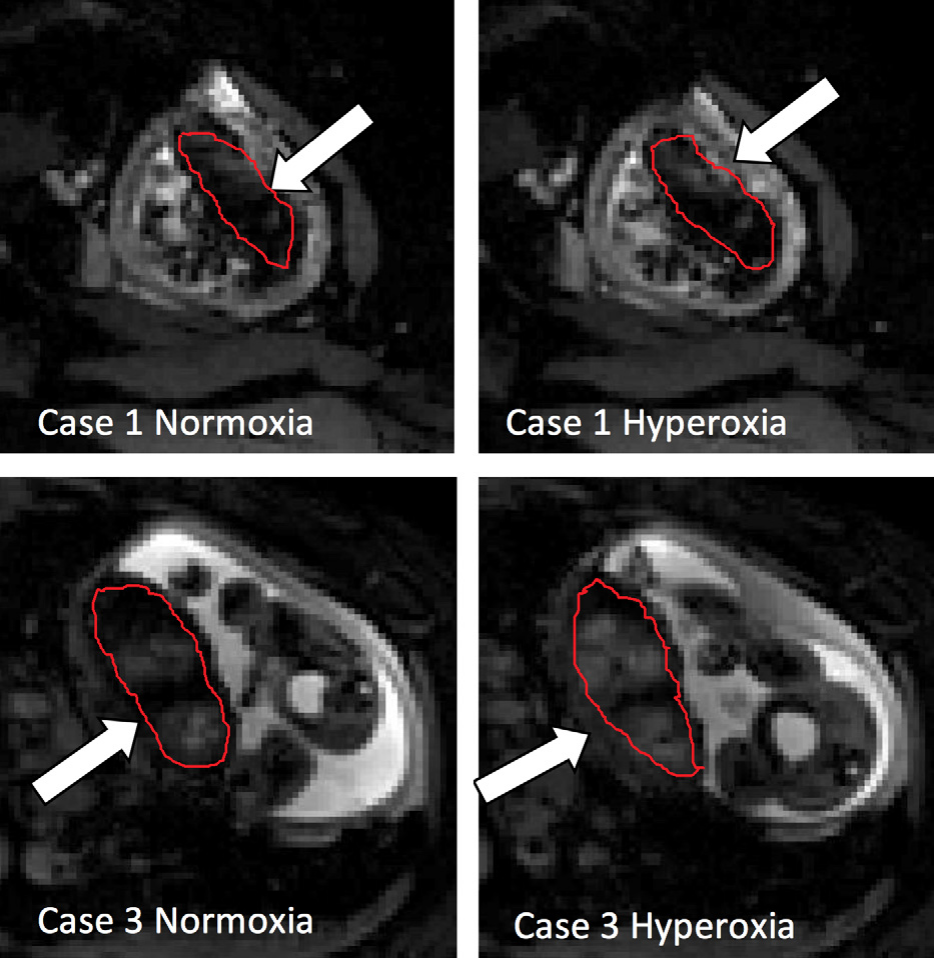
Placental BOLD images obtained during maternal normoxia and hyperoxia. Placenta is marked with a white arrow. Case 1 (image A1 and A2): Non-responder. Case 3 (image B1 and B2): Hyperresponder.

The placental BOLD response can be evaluated visually, as the placenta appears brighter in the BOLD image when the oxygenation is increased. BOLD images of Case 1 (nonresponder) and Case 3 (hyper-responder) is demonstrated in normoxic and hyperoxic phase in Figure2.

**Figure 3:**
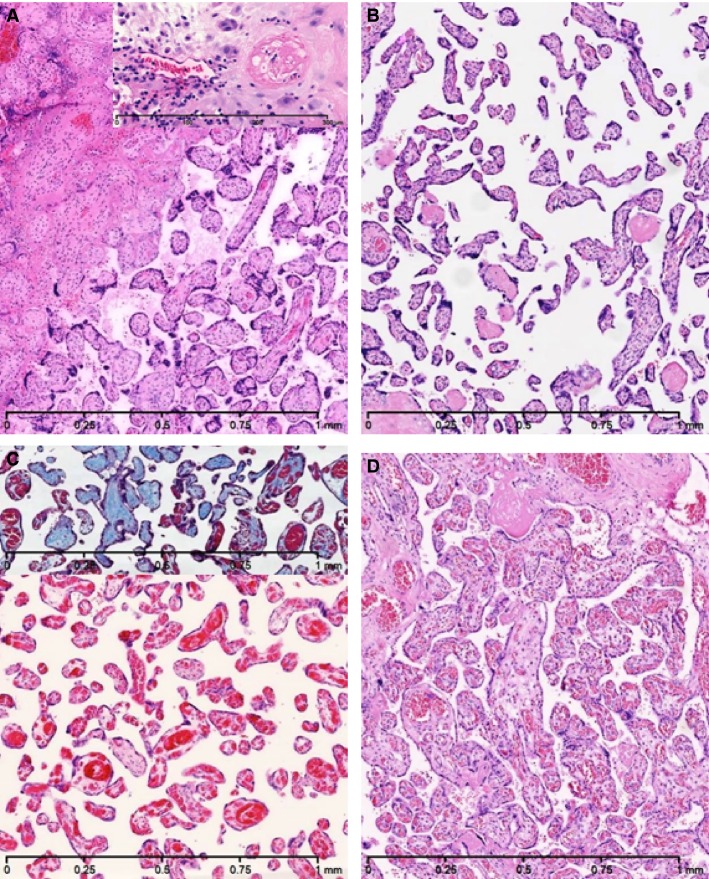
Placental histology (original magnification 50X). Haematoxylin-eosin staining except the inset in Figure (C) which is Masson’s Trichrome staining. (A) Case 1: Infarct (left) and accelarated villous maturation (right). Inset: Normal maternal decidual artery (left) and maternal decidual vasculopathy (right). (B) Case 2: Accelarated villous maturation and distal villous hypoplasia. (C) Case 3: Accelated villous maturation and distal villous hypoplasia. Inset: Fetal thrombotic vasculopathy with fibrous avascular villi (blue). (D) Case 4: Normal villi.

The histological placental findings of the four FGR cases is demonstrated in Figure3. In Case 1, 2, and 3 the placental examination revealed abnormalities associated with maternal hypoperfusion such as; low placental weight, distal villous hypoplasia, accelerated villous maturation, and varying degree of placental infarctions (given as a percentage of the total placental parenchyma); Case 1: 65%, Case 2: 50%, and Case 3: 5%. In addition, in Case 1, increased intervillous fibrin deposition and maternal decidual vasculopathy was demonstrated, and Case 3 revealed fetal thrombotic vasculopathy (small groups of fibrous avascular villi) as a result of a marginal umbilical cord insertion. In Case 4, the placental examination demonstrated low placental weight, however, the placental histology was normal.

## Discussion

In this study, the hyperoxic placental BOLD response was investigated in 21 normal pregnancies and in four cases of FGR. In the normal group, we demonstrated a significant increase in the placental BOLD signal during maternal hyperoxia. In FGR pregnancies, an abnormal BOLD response was demonstrated only in cases with histological signs of maternal hypoperfusion of the placenta.

A limitation of this study is the small number of FGR cases. This case report focusing on individual characteristics is supposed to precede larger studies focusing on group differences. However, the novelty of this method for estimating placental oxygen transport capacity makes this article highly relevant. The lack of histological placental examination in the normal group of pregnancies is another limitation of this study. However, in the normal group, placenta is assumed normal as all fetuses had normal birth weight and normal Doppler findings at the time of the MRI examination. Furthermore, a limitation of this study is that there is a time interval between the placental BOLD MRI and the postpartum placental examination. In other placental MRI studies, it has been demonstrated that the correlation between placental MRI findings and postpartum placental examination decreases with increasing time interval between the two (Derwig et al. 2013).

Strength of this study is that the FGR cases are in-depth characterized by Doppler flow measurements and placental histology. In studies of FGR pregnancy, it is important to identify the true FGR cases by abnormal ultrasound Doppler flow measurements and to confirm the diagnosis by placental histological examination postpartum. If the FGR cases are identified only by low birth weight, which is often the case in larger studies, normal small fetuses will be included in the FGR group.

As previously stated in this article, the hyperoxic placental BOLD response is derived mainly from an increase in the saturation of the maternal venous blood of the placenta. In the placenta, the pO_2_ of the fetal umbilical vein is closely related to the pO_2_ of the uterine vein, therefore the hyperoxic placental BOLD response reflects the increase in the fetal oxygen supply provided by maternal hyperoxia. Previous BOLD MRI studies in the sheep fetus (Sorensen et al. 2009) and the human pregnancy (Sorensen et al. 2013a,b) have demonstrated that maternal hyperoxia increases fetal and placental oxygenation. This can be explained by an increase in the partial pressure gradient of oxygen across the placental membrane. In uterus, the fetus lives in a relative hypoxic environment (Martin et al. 2010), and the analogy of “Everest in Utero” was mentioned by Sir Joseph Barcroft more than 70 years ago. The fetal umbilical vein blood is not fully saturated at room air, and therefore the increase in umbilical vein pO_2_ increases the saturation of fetal blood and thereby fetal oxygenation (Jackson et al. 1987).

Apart from increases in tissue oxygenation, other factors such as the tissue blood volume and the orientation of the blood flow in the placenta might affect the baseline BOLD signal and to a lesser extent, the hyperoxic BOLD response. These factors might be altered in the FGR cases when compared to the normal group. Even though, the histopathological placental examination of the FGR cases did not reveal such findings, any contribution of such factors on the MRI findings cannot be excluded.

In our study, an abnormal hyperoxic BOLD response was demonstrated in three out of four FGR cases, namely the three FGR cases in which the placental histology revealed signs of maternal hypoperfusion. In FGR Case 2 and 3, the BOLD response was increased above normal (hyper-responders). This finding may indicate that the placental oxygen transport capacity was intact, as the hyper-response could be explained by a reduced baseline placenta oxygenation in FGR pregnancies due to maternal hypoperfusion. Therefore, any given increase in absolute BOLD signal leads to a larger increase in the relative BOLD response (ΔBOLD) when baseline is reduced. In Case 1, the placental BOLD signal was not increased during hyperoxia (nonresponder). This finding may reflect a severely impaired placental oxygen transport, as maternal perfusion is severely reduced. This suggestion is supported by the histological findings of massive placental infarction and decidual vasculopathy. We hypothesize that the abnormal placental BOLD response of these FGR cases is a continuum; initially, the response is increased because of the initial placental hypoxia. However, as the placental pathology advances, the BOLD response is reduced because of the reduced placental oxygen transport.

In our study, no increase in placental the BOLD signal (Case 1) was associated with adverse neonatal outcome. In this case, cesarean section on fetal indications was never an option because of very low estimated fetal weight, and unfortunately stillbirth occurred in gestational week 26 + 1 day. The neonatal outcome was good in the remaining three FGR Cases; Case 2 and 3 had emergency cesarean sections because of non-reassuring fetal heart rate pattern in gestational week 29 and 31, respectively, and case 4 had an elective cesarean section at gestational week 34.

Previous studies have investigated negative predictive value of maternal hyperoxia. In one study, the fetal effect of maternal hyperoxia was estimated by cordocentesis, and no increase in umbilical pO_2_ was associated with adverse neonatal outcome (Nicolaides et al. 1987). Another study estimated the changes in fetal cerebral blood flow during maternal hyperoxia, and no change in fetal cerebral blood flow was associated with poor neonatal outcome (Arduini et al. 1988). In each of these studies, being a non-responder to maternal hyperoxia was associated with impaired placental oxygen transport and the need of immediate delivery.

In early onset FGR, the placental oxygen transport is limited mainly by maternal perfusion (Baschat 2004; Mifsud and Sebire 2014), however, in some FGR cases, in addition, the diffusion of oxygen across the placental membrane might be impaired due to increased membrane thickness and reduced surface area (Macara et al. 1996). In these FGR placentas, the pO_2_ of the umbilical vein remains markedly lower than the pO_2_ of the maternal uterine vein. In such cases, the hyperoxic BOLD response which is mainly derived from the oxygenation changes of the uterine vein tends to overestimate the fetal oxygen supply. However, a hyperoxic placental BOLD response below normal may suggest an impaired fetal oxygen supply in any FGR case.

According to our knowledge, this is the very first study to investigate the placental oxygen transport by placental BOLD MRI in human FGR pregnancy. However, previously the hyperoxic BOLD response has been investigated in a rat model of FGR (Aimot-Macron et al. 2013). In the rat model, the uterine vascular pedicle was ligated on the left side of the bicornuate uterus, leading to FGR due to maternal hypoperfusion. The hyperoxic BOLD response of the feto-placenta unit was significantly reduced in the ligated horn when compared to the normal horn. These findings are in accordance with our findings of Case 1, in which the placental BOLD response is reduced due to impaired placental oxygen transport. In less severe cases, the BOLD response most likely would have been increased.

In our study, we found that in the four FGR cases, the placental histology was closely related to the Doppler flow findings. The degree of placental pathology was associated with the degree of fetal redistribution of blood flow which is an indicator of fetal hypoxia and acidosis (Baschat 2005). The Doppler flow examination of the uterine arteries was abnormal only in FGR cases with histological signs of maternal hypoperfusion. This finding is expected, as the Doppler flow of the uterine arteries reflects the resistance of the spiral arteries (Lin et al. 1995). However, the degree of placental pathology was not directly related to the uterine artery PI. In the four FGR cases, the hyperoxic placental BOLD response was associated with the degree of fetal redistribution as well as the placental histology. A direct comparison between the fetal Doppler findings and the placental BOLD response would need more FGR cases. However, investigating the placental function rather than fetal distress should give us an opportunity to discover the potential placental problems at an earlier stage.

This is the very first study investigating the human placental oxygenation transport by the hyperoxic placental BOLD response in normal and FGR pregnancy. In FGR cases, an abnormal hyperoxic placental BOLD response was demonstrated only in the three FGR cases with histological signs of maternal hypoperfusion. This pilot study suggests that the placental BOLD MRI might provide us important information regarding the placental oxygen transport in vivo. Further research is needed in this field.
